# Dynamics of Cellular Plasticity in Prostate Cancer Progression

**DOI:** 10.3389/fmolb.2020.00130

**Published:** 2020-07-10

**Authors:** Ritika Tiwari, Nishat Manzar, Bushra Ateeq

**Affiliations:** Molecular Oncology Laboratory, Department of Biological Sciences and Bioengineering, Indian Institute of Technology Kanpur, Kanpur, India

**Keywords:** prostate cancer, ADT, cellular plasticity, EMT, stemness, drug resistance, NEPC

## Abstract

Despite the current advances in the treatment for prostate cancer, the patients often develop resistance to the conventional therapeutic interventions. Therapy-induced drug resistance and tumor progression have been associated with cellular plasticity acquired due to reprogramming at the molecular and phenotypic levels. The plasticity of the tumor cells is mainly governed by two factors: cell-intrinsic and cell-extrinsic. The cell-intrinsic factors involve alteration in the genetic or epigenetic regulators, while cell-extrinsic factors include microenvironmental cues and drug-induced selective pressure. Epithelial-mesenchymal transition (EMT) and stemness are two important hallmarks that dictate cellular plasticity in multiple cancer types including prostate. Emerging evidence has also pinpointed the role of tumor cell plasticity in driving anti-androgen induced neuroendocrine prostate cancer (NEPC), a lethal and therapy-resistant subtype. In this review, we discuss the role of cellular plasticity manifested due to genetic, epigenetic alterations and cues from the tumor microenvironment, and their role in driving therapy resistant prostate cancer.

## Introduction

Prostate cancer (PCa) represents a highly heterogenous disease with diverse range of molecular alterations defining its subclasses. These molecular alterations include somatic or germline mutations, focal deletions, amplifications, and gene fusions that entail the intra- and inter-patient heterogeneity and confer variable clinical outcomes. The major molecular subclasses include a variety of gene fusions involving *ETS* family transcription factors, namely *ERG, ETV1/4, FLI1*, and *NDRG1*; or *RAF* kinase rearrangements, upregulation of secretory protein SPINK1 and somatic mutations in *SPOP, FOXA1*, and *IDH1* (Tomlins et al., 2005, 2008; Palanisamy et al., 2010; Cancer Genome Atlas Research, 2015; Bhatia and Ateeq, 2019). The androgen signaling plays a key role in development and maintenance of the prostate gland (Cunha et al., 1987; Cooke et al., 1991), while aberrant activation of this signaling has been linked to the initiation and metastatic progression of PCa (Gelmann, 2002; Culig and Santer, 2014; Tan et al., 2015). Thus, drugs that target biosynthesis of androgen or androgen receptor (AR) activity are often administered as the first line therapy also known as androgen deprivation therapy (ADT). However, the disease inadvertently progresses to an advanced stage, castrate-resistant prostate cancer (CRPC) (Cher et al., 1996; Gregory et al., 2001; Chen et al., 2004; Grasso et al., 2012; Robinson et al., 2015). At CRPC stage, the cancer cells bypass their dependency on the androgen signaling by various mechanisms such as somatic mutations or amplification of *AR* gene, constitutively active splice variants (AR-V7 and ARv567es), mutations in the ligand binding domain of AR (F877L and T878A), or activation of androgen-regulated genes via glucocorticoid receptor (Taplin et al., 1995; Arora et al., 2013; Antonarakis et al., 2014). The androgen biosynthesis inhibitor, abiraterone and next-generation AR-antagonists, enzalutamide and apalutamide have been developed for the clinic management of CRPC patients (Scher et al., 2010; de Bono et al., 2011; Clegg et al., 2012). Although, AR-targeting therapies prolong the overall survival of the patients, nonetheless, resistance to these drugs often prevail leading to disease progression to an aggressive stage, also known as neuroendocrine prostate cancer (NEPC) (Aggarwal et al., 2018). The mechanism to overcome the acquired resistance toward anti-androgen therapy is frequently manifested by several molecular and phenotypic changes resulting in transition of androgen-independent CRPC to therapy-induced NEPC (Zou et al., 2017; Aggarwal et al., 2018; Soundararajan et al., 2018; Beltran et al., 2019). This dynamic transition provides multifaceted advantages to the cancer cells to overcome therapy-induced resistance and enable survival (Sun et al., 2012; Miao et al., 2017; Stylianou et al., 2019).

Cellular plasticity represents the dynamic transition of a cell between one state to another (Varga and Greten, 2017). The term “plasticity” was introduced to define the extensive reprogramming events happening in stem cells leading to cellular differentiation (Blau et al., 1985). This is a bidirectional process which involves changes both at the molecular and phenotypic levels of a cell. The cellular plasticity has been a key phenomenon that governs not only the developmental fate of the organism, but also serves as a driving force behind different malignancies, including PCa (Rothman and Jarriault, 2019; Yuan et al., 2019). During early embryonic development, the cellular plasticity helps the stem or progenitor cells to differentiate into different lineages while in the later stages of life, it maintains stem cell populations and regulates tissue repair (Wagers and Weissman, 2004). Several complex processes such as transcriptional regulation or epigenetic alterations are known to modulate the cellular identity and plasticity (Flavahan et al., 2017). Mounting evidence suggests that the genes involved in the embryonic development are frequently subverted or reactivated during malignant transformation of cells (Kalluri and Weinberg, 2009; Dempke et al., 2017). These acquired molecular attributes enable the tumor cells to elude the constraints of normal growth, thereby assisting them to thrive and sustain, escape therapeutic pressure and immune surveillance (Zou et al., 2017; Vitkin et al., 2019; Yuan et al., 2019). Likewise, in PCa, cellular plasticity aids the tumor cells to develop resistance against the targeted therapies in several different ways, for instance, by undergoing phenotypic conversions, cellular reprogramming and transition from one cell lineage to another (Beltran and Demichelis, 2015; Zou et al., 2017; Alumkal et al., 2020).

In this review, we discuss the importance of cellular plasticity in conferring intra-tumoral heterogeneity and its impact on disease progression and drug resistance. Further, we attempt to delineate the implications of cell-intrinsic and -extrinsic factors which govern the plasticity in tumor cells. Finally, we also summarize the novel therapeutic interventions used to target cellular plasticity in combating prostate cancer.

## Intra-Tumoral Heterogeneity and Cellular Plasticity

PCa exhibits high level of intra-tumor heterogeneity characterized by distinct sub-populations of the cancer cells, which is often a major confounding factor influencing disease progression (Boyd et al., 2012; Yadav et al., 2018). This intra-tumor heterogeneity offers a multifaceted advantage to the PCa cells such as disease progression, tumor dissemination, and driving resistance toward standard therapies such as chemotherapy, radiation or hormone therapy (Marjanovic et al., 2013). Two different models contributing to intra-tumoral heterogeneity in PCa have been generally accepted. In the clonal evolution model, tumors arise from a single cell of origin triggered in response to sequential oncogenic hits (Liu et al., 2009; Kreso and Dick, 2014). In cancer stem cell model, tumor cells originate from the differentiation of a small population of cancer stem cells (CSCs) or dedifferentiation of the existing cancer cells into CSCs to promote tumor growth and progression (Collins et al., 2005; Patrawala et al., 2006; Yadav et al., 2018). The neoplastic transformation via either of the proposed pathways give rise to genetically and phenotypically distinct cell types within same tumor (Poli et al., 2018). This morphological heterogeneity is responsible for the multifocality within the prostate tumor of the same patient. The multifocality has been reported in ~50–90% of the PCa patients who underwent radical prostatectomy, and has been linked with higher grade, advanced stage and recurrence compared to unifocal prostate adenocarcinoma (Djavan et al., 1999). Multifocal tumors exhibit significant molecular heterogeneity in terms of copy number alterations (CNAs), single-nucleotide variants (SNVs), genomic rearrangements, and unique signatures of DNA damage and transcriptional dysregulation (Beltran and Demichelis, 2015; Boutros et al., 2015). Additionally, intra-tumoral variability involving distinct DNA methylation and histone modification patterns was found to be more pronounced in the advanced stage PCa, suggesting association of epigenetic heterogeneity with poor clinical outcome (Seligson et al., 2005; Bianco-Miotto et al., 2010; Brocks et al., 2014). Thus, deciphering the molecular basis of the intra-tumor heterogeneity may provide an insight for better prognosis and the clinical management of PCa patients.

## Epithelial-Mesenchymal Transition Modulates Cellular Plasticity in Prostate Cancer

The epithelial-mesenchymal transition (EMT) is the key phenomenon in embryonic development, nonetheless, it plays a pivotal role in maintaining tissue homeostasis as well as cancer progression (Nauseef and Henry, 2011). This complex process involves transition of a epithelial cell into a mesenchymal phenotype, characterized by reduced cell-cell adhesion and increased migratory properties (Lu and Kang, 2019). Moreover, tumors often exhibit co-existence of a subpopulation of cells in hybrid state harboring both epithelial and mesenchymal phenotypes (hybrid E/M state), further aiding the cancer cells to metastasize from primary to distant secondary sites (Tsai and Yang, 2013; Williams et al., 2019). During this reprogramming, the cancer cells secrete an array of enzymes which break down its attachment to the basement membrane followed by several phenotypic changes such as reorganization of actin cytoskeleton, leading to enhanced migratory and metastatic potential (Thiery et al., 2009). Multiple clinical evidence has associated enhanced mesenchymal features with high Gleason grade, shorter time to biochemical recurrence and increased metastasis in PCa (Cheng et al., 1996; Graham et al., 2008; Zhang et al., 2009; Figiel et al., 2017).

Several transcription factors (TFs) associated with EMT regulate cellular plasticity during embryonic development have been identified as the oncogenic determinants in the neoplastic transformation of prostate. For example, SRY-Box Transcription Factor 9 (SOX9) enables transition of fetal prostate epithelial cells into mesenchyme during embryogenesis and its high levels in advanced stage PCa has also been reported (Wang et al., 2008). Furthermore, Wnt/β-catenin signaling which is linked with the initiation and progression of multiple cancers is also known to regulate expression of Sox9 (Clevers, 2006). Another key feature of EMT is the loss of adherens junction protein, E-Cadherin (E-Cad), a tumor suppressor required for maintaining the epithelial phenotype (Loh et al., 2019). Moreover, downregulation of E-Cad via Notch signaling is also known to promote drug resistance in PCa cells (Wang et al., 2017). In addition, zinc finger proteins belonging to Snail family transcriptional repressors, SNAI1 (SNAIL) and SNAI2 (SLUG), and zinc finger E-box-binding homeobox 1 and 2 (ZEB1 and ZEB2) and Twist-related protein 1 (TWIST1) are the key TFs involved in EMT, which also downregulate E-Cad and upregulate various mesenchymal markers, namely N-Cadherin (N-Cad), Vimentin (VIM) and Fibronectin (Jennbacken et al., 2010; Zhu and Kyprianou, 2010; Sun et al., 2012; Shiota et al., 2014; Zhifang et al., 2015; Miao et al., 2017). In a recent study, a positive feedback loop has been demonstrated between SOX4 and a scaffold protein Cullin 4B (CUL4B), wherein CUL4B induces the SOX4 expression via PRC2-mediated silencing of miR-204 and in turn SOX4 positively regulates the transcription of CUL4B, leading to enhanced proliferation and invasion of PCa cells. In addition, the CUL4B+/SOX4+ subset of PCa patients show activation of Wnt/β-catenin signaling pathway and are associated with an aggressive disease and poor prognosis (Qi et al., 2019).

In PCa, the selection pressure imposed by ADT has been well-known to potentiate EMT and stemness (Sun et al., 2012; Tsai et al., 2018). Importantly, androgen deprivation in mice implanted with human LuCaP35 prostate tumor induces the increased expression of *N-Cadherin* (*CDH2*), *VIM, ZEB1, TWIST1*, and *SLUG*. Notably, a bidirectional negative feedback loop is generated between AR and Zeb1 which is involved in androgen deprivation induced EMT (Sun et al., 2012). Moreover, LNCaP cells treated with epigenetic drugs lead to upregulation of *ZEB1* and reduced AR levels, whereas siRNA mediated *ZEB1* silencing leads to increased expression of AR (Sun et al., 2012). Interestingly, enhanced expression of *ZEB1* due to copy number gain leads to direct transcriptional repression of miR-33a-5p in PCa cells, and contribute to an increase in EMT, invasion, migration and bone metastasis (Dai et al., 2019). Besides, miR-33a-5p indirectly inhibits ZEB1 expression via targeting TGFBR1 and suppressing TGF-β signaling, thus forming an indirect double-negative feedback loop. AR is also known to act as the direct transcriptional repressor of SNAIL, and its upregulation along with ZEB1/2, TWIST and Forkhead box protein C2 (FOXC2) has been reported as an adaptive response to androgen deprivation (Miao et al., 2017). Intriguingly, tumor grafts derived from PCa patients who underwent radical prostatectomy following neoadjuvant ADT (6–8 weeks of flutamide or lupron) exhibit mislocalization of E-Cad and elevated VIM expression (Zhao et al., 2013).

Conversely, ZEB2, another critical mediator of EMT shows AR mediated differential regulation in androgen dependent vs. independent manner. In androgen-dependent LNCaP cells, ZEB2 is positively regulated by AR and showed increased expression upon androgen stimulation while reduced expression in *AR*-silenced cells. In androgen-independent cell lines, such as PC3 and DU145, ectopic expression of AR leads to upregulation of miR-200a/miR-200b resulting in reduced expression of ZEB2 accompanied with diminished invasive potential (Jacob et al., 2014). This context-dependent AR mediated regulation of ZEB2 may be due to the differences in the levels and types of co-regulatory proteins which modulate AR activity as an activator or repressor (Van De Wijngaart et al., 2012). In another study, miR-145 has been shown to post-transcriptionally suppress the expression of ZEB2 resulting in decreased invasion, migration and stemness in PCa cells (Ren et al., 2014). Moreover, ZEB2 acts as a direct transcriptional repressor of miR-145 and its downregulation in PC3 cells results in reduced bone invasion in mouse models, suggesting a double-negative feedback loop between ZEB2 and miR-145. Unlike SNAIL, which is an AR repressed gene, the SLUG expression was found to be upregulated by constitutively active AR signaling in a ligand-independent manner. Additionally, SLUG also serves as a novel co-activator of AR and enhances its transcriptional activity even in the absence of androgens (Wu et al., 2012). Another study has shown that siRNA-mediated *AR* silencing in PCa cells promoted migration and invasion via C-C motif chemokine ligand 2 (CCL2)-dependent STAT3 activation and subsequent upregulation of EMT associated pathways (Izumi et al., 2013). In a follow-up study, targeting pSTAT3–CCL2 signaling with C-C chemokine receptor type 2 (CCR2) antagonists reversed the ADT induced cell invasion and macrophage infiltration in transgenic adenocarcinoma of the mouse prostate-C1 (TRAMP-C1) mouse tumors (Lin et al., 2013). One possible explanation could be that AR is known to directly regulate SPDEF (SAM pointed domain-containing ETS transcription factor), a transcriptional repressor of *CCL2*, and ADT leads to reduced SPDEF expression resulting in elevated CCL2 levels (Tsai et al., 2018). Thus, the importance of AR-signaling in EMT is context-dependent in PCa and needs to be further delineated in order to understand the pathobiology of this disease and develop effective therapeutic approaches.

While EMT helps with the initial dissemination of the tumor cells, clinical manifestation of the metastases depends upon mesenchymal-epithelial transition (MET), which is crucial for the effective seeding and colonization of the disseminated tumor cells at the distant metastatic site (Nieto, 2013). For instance, the cross-talk between the metastasized PCa cells and stroma in liver show elevated expression of the E-Cad, possibly due to MET induced cellular plasticity (Yates et al., 2007). This dynamic transition through a spectrum of phenotypically different states could potentially regulate the initial dissemination of PCa cells followed by metastatic spread to the distant sites. However, more evidence is required to support the notion of EMT-MET axis in cellular reprogramming and may serve as a promising therapeutic strategy in targeting disease progression in prostate cancer.

## Stemness Imparts Cellular Plasticity in Prostate Cancer

The CSCs constitute a small population of tumor cells which has the potential to drive cancer progression, increased resistance to conventional therapies and ability to disseminate to distant organs (Soundararajan et al., 2018; Li and Shen, 2019). However, the theory about the exact origin of CSCs is still debatable. It has been suggested that CSCs are either derived directly from the normal stem cells or produced as a result of de-differentiation or trans-differentiation of the existing cancer cells (Friedmann-Morvinski and Verma, 2014; Plaks et al., 2015).

The role of EMT in imparting stemness is much in contrast to its significance in the normal embryonic development, wherein it primarily governs the differentiation of stem cells into multiple lineages (Wang and Unternaehrer, 2019). EMT promoting transcription factors, such as ZEB1 is known to promote stemness in PCa (Wellner et al., 2009; Orellana-Serradell et al., 2018). Moreover, ectopic expression of platelet-derived growth factor D (PDGF-D) in PC3 cells lead to morphological changes associated with acquisition of EMT and increased clonogenicity and sphere-forming abilities. These cells also show enhanced expression of TFs associated with stemness such as Nanog, Oct4 and Sox2, Lin28B and members of polycomb repressor complex 2 (PRC2) (Kong et al., 2010). Moreover, human PCa derived LuCaP35 xenografts when subjected to ADT show concomitant higher expression of EMT as well as stem cell markers, namely WNT5a and WNT5b (Sun et al., 2012). Although EMT is known to promote tumorigenesis, a subpopulation of tumor cells with epithelial phenotype are reported to have high metastatic potential (Celia-Terrassa et al., 2012). Also, cells undergoing EMT have increased invasive ability but diminished capacity of establishing distant metastasis (Tsuji et al., 2008; Floor et al., 2011). Furthermore, it has been demonstrated that a subpopulation of cells with epithelial phenotype and high E-Cad expression, also shows enhanced stemness and self-renewal ability (Celia-Terrassa et al., 2012). Intriguingly, a recent study has shown the tumor promoting role of E-Cad in invasive ductal carcinomas of breast, wherein it promotes tumor growth and metastases (Padmanaban et al., 2019). However, E-Cad has been implicated majorly as a tumor-suppressor across multiple cancer types, and its loss is directly involved in imparting various oncogenic traits especially stemness and metastases (Frixen et al., 1991; Berx et al., 1995; Guilford, 1999; Onder et al., 2008).

The CSCs express a broad range of cell surface markers which distinguish them from the cells of other origins. For instance, prostate CSCs (PCSCs) harbor expression of several cell surface markers such as CD44^+^α2β1^hi^CD133^+^ (Collins et al., 2005). The CD44^+^ cell population derived from multiple PCa cell lines and xenograft tumors showed increased tumorigenic and metastatic potential along with enhanced expression of stemness promoting TFs factors namely, Oct-3/4, Bmi and β-catenin (Patrawala et al., 2006). The CD44 is considered as a putative marker for PCSCs and primarily expressed on the surface of basal and rare neuroendocrine cells, whereas the luminal cells lack its expression (Palapattu et al., 2009; Wang and Shen, 2011; Guo et al., 2012). The pluripotent basal cells differentiate to luminal and neuroendocrine cells, and hence been proposed to have high tumorigenic potential and could serve as cells of origin in prostate carcinogenesis (Goldstein et al., 2010; Taylor et al., 2012). It has also been reported that luminal multilineage progenitor cells are the cells of origin and basal cells transition to luminal cells in order to promote tumorigenesis (Karthaus et al., 2014; Wang et al., 2014). Further, ABCG2, a well-known ATP-binding cassette transporter (ABC transporter) associated with drug efflux is known to be highly expressed in PCSCs and drives resistance to therapeutic agents (Huss et al., 2005).

Similar to EMT, the dedifferentiated PCSCs show inverse correlation with AR signaling. For instance, PSA^−/lo^ (PSA-negative or low) cell population exhibits gene expression profile similar to stem cells, harbors enhanced self-renewing potential and resistance to ADT and chemotherapeutic agents (Qin et al., 2012). The PCSCs isolated from AR-negative DU145 cells show higher expression of CD44, CD24, integrin α2β1, cellular reprogramming factor SOX2, and exhibit tumor-initiating potential and self-renewal ability (Rybak et al., 2011; Rybak and Tang, 2013). Similarly, a subpopulation of tumor cells isolated from prostatectomy specimens express higher levels of tumor-associated calcium signal transducer 2 (Trop2), CD44, and CD49f, and show increased sphere-forming ability and regeneration capability in mice (Garraway et al., 2010). Conclusively, a consensus regarding a specific set of markers to identify PCSCs is still lacking and in-depth study is warranted to identify the defined markers for multipotent tumor progenitor cells in order to develop better therapeutic strategies.

## Therapy-Induced Cellular Plasticity and Disease Progression

The cancer cells evade the drug induced therapeutic pressure by modulating cellular plasticity which is one of the major mechanisms posing significant challenges for PCa treatment (Boumahdi and de Sauvage, 2019). The plasticity of the tumor cells provides a survival advantage by developing alternate adaptive pathways, independent of the targeted therapies. As mentioned previously, ADT is administered as the standard care for the treatment of men with prostate cancer. One of the main mechanisms of eluding AR-targeted therapy or ADT is the transdifferentiation of the AR-dependent PCa cells to AR-independent neuroendocrine (NE)-like phenotype (Lin et al., 2014). Transdifferentiation is a process wherein a differentiated cell type transitions to another lineage to evade the therapy-associated drug pressure (Davies et al., 2018).

This transition process in response to therapy is often driven by a distinct transcriptional or epigenetic reprogramming of the tumor cells (Yuan et al., 2019). Recent evidence highlighted the role of EMT and stemness as important driving factors for the cellular plasticity during the neuroendocrine transdifferentiation (Soundararajan et al., 2018). Several transcription factors which are directly involved in regulating EMT are also key players involved in neuroendocrine transdifferentiation. For instance, overexpression of SNAIL imparts cellular plasticity by downregulating the E-Cad expression and enhancing the expression of neuroendocrine differentiation markers, namely, ENO2 and CHGA (McKeithen et al., 2010; Barnett et al., 2011). Similarly, neuroendocrine transdifferentiation of patient-derived LTL331 xenograft model also exhibits higher levels of SNAI1 and ZEB1 (Akamatsu et al., 2015).

The PCa cells have the ability to dedifferentiate into CSCs exhibiting tumor-initiating potential with an invasive phenotype and resistance to AR-antagonists. These reprogrammed cells when exposed to androgens in culture showed reactivation of the AR signaling, indicating the active dynamics of the cellular plasticity in response to the external cues (Nouri et al., 2017). The advanced neuroendocrine tumors such as small cell NE-like carcinomas are often characterized to have stem cell-like features (Ellis and Loda, 2015). Moreover, pluripotency factors, SOX2 and SOX11 have also been implicated in AR-independent NE-like tumors (Blee and Huang, 2019). Recent evidence suggested that BRN2 co-regulates the transcriptional landscape of the SOX2 and is essentially overexpressed in NEPC patients (Bishop et al., 2017). The elevated levels of EMT modulator ZEB1 also induces stem-cell like properties in PCa cells along with concomitant upregulation of SOX2 (Li et al., 2014). Apart from the critical role of EMT and CSCs in evading therapeutic pressure, several inherent factors also play an important role in imparting resistance to the therapy. For example, genetically engineered mouse (GEM) model with inactivation of Pten and Tp53 failed to show any response to abiraterone, and exhibited accelerated progression to treatment-induced neuroendocrine differentiation (Zou et al., 2017). A recent single-arm enzalutamide clinical trial revealed that non-responders to enzalutamide treatment exhibits a basal lineage, such as reduced AR transcriptional activity and a neurogenic/stemness program, while a luminal lineage program was activated in responders (Alumkal et al., 2020), indicating that there is need to explore the specific factors that regulate *de novo* enzalutamide resistance.

## Factors Governing Cellular Plasticity

### Cell-Intrinsic Factors

In the past decade, multiple independent studies unraveled the diverse spectrum of molecular and other environmental factors governing the PCa lineage plasticity. Dramatic differences in the gene expression and copy number alterations has been reported to co-exist between the prostate adenocarcinoma and the NEPC, often within the same tumor foci (Beltran et al., 2011). Moreover, comprehensive molecular characterization of the NEPC tumors revealed the significance of divergent clonal evolution. Under the influence of therapy, CRPC cells give rise to new clones owing to epithelial plasticity, with distinct molecular profiles and genetic aberrations. Initially, few molecular alterations occur that drive and select clones for the cellular plasticity, followed by a series of passenger alterations which may result in the emergence of therapy-resistance NEPC (Beltran et al., 2016).

Most of the prostatic small cell carcinomas (SCC) harbor the *TMPRSS2-ERG* gene rearrangement confirming its involvement in the carcinogenesis. Although, *TMPRSS2-ERG* fusion is not reported in the SCC of non-prostatic origins, such as lung and urinary bladder, indicating that this genetic event can be used as a molecular marker to establish the prostatic origin of metastatic SCC (Guo et al., 2011). Of note, NEPC foci often lack the expression of ERG protein in the tumors harboring *TMPRSS2-ERG* fusion, which reaffirms the reduced or absent androgen signaling. A classic example of this ambiguity is the NEPC cell line model, NCI-H660 which harbors *TMPRSS2-ERG* fusion, but lacks expression of ERG protein (Beltran et al., 2011). Furthermore, ERG oncoprotein suppresses the expression of NEPC related genes in PCa which is relieved upon inhibition of AR signaling (Mounir et al., 2015).

The mutational landscape of NEPC patients has identified the role of *RB1* loss and mutated/deleted *TP53* in the SCC pathogenesis. In contrast to the CRPC-adenocarcinoma patients, CRPC-NE patients showed reduced frequency of genomic alterations associated with androgen receptor (AR), indicating the selection of AR-independent clonal subpopulation during NEPC progression (Beltran et al., 2016). Simultaneous aberration in various tumor suppressor genes (*RB1, TP53*, and/or *PTEN*) has been known to drive tumor plasticity in PCa (Aparicio et al., 2016). For instance, knockdown of *TP53* and *RB1* using short hairpin RNAs (shRNAs) in AR overexpressing LNCaP cells resulted in the enhanced expression of basal and neuroendocrine lineage markers thereby conferring resistance to anti-androgen therapy (Aparicio et al., 2016).

Overexpression of Aurora kinase A (AURKA) and oncogene N-Myc (MYCN) due to gene amplification was found in NEPC cases, where both proteins cooperate in driving the NE-transdifferentiation. Although, being located on different chromosomes, the mechanism involved in their co-amplification in NEPC remains unknown, but certainly hints toward their usefulness as diagnostic markers for early intervention in the high-risk population (Beltran et al., 2011). Interestingly, this discovery formed the basis to use Aurora kinase A inhibitors for the treatment of NEPC patients harboring AURKA amplification (Beltran et al., 2019). Moreover, activated AKT1 and MYCN are also known to drive the transformation of prostate epithelial cells to adenocarcinoma and differentiation to NE-like phenotype (Lee et al., 2016). MYCN in cooperation with PRC2 complex member, EZH2 and other cofactors suppress the AR signaling and PRC2 target genes (Beltran et al., 2011; Dardenne et al., 2016; Lee et al., 2016). Apart from EZH2, other PRC1 containing proteins, such as members of CBX family have also been shown to be dysregulated in patient-tumor derived xenografts (PDX) and NEPC clinical samples, highlighting a role for dysregulated Polycomb Group (PcG)-mediated silencing during NE-transdifferentiation (Clermont et al., 2015).

Reduced expression of RE1 silencing transcription factor (REST), a master negative regulator of neuroendocrine differentiation accompanied with enrichment of the REST target NE-associated genes has been reported in the NEPC clinical samples (Lapuk et al., 2012). Interestingly, another member of REST transcriptional repressor complex, PHD finger protein 21A (PHF21A) is differentially spliced in NEPC cases compared to adenocarcinoma. PHDF21A loses the AT-hook domain which is involved in the DNA binding via alternative splicing (Lapuk et al., 2012). In LNCaP cells, androgen stimulation leads to co-occupancy of REST on the AR occupied chromatin regions and mediates transcriptional repression of a subset of genes. Further, siRNA mediated *REST* silencing leads to upregulation of genes associated with neuronal differentiation and maintenance of NE phenotype (Svensson et al., 2013). Moreover, activation of androgen signaling enhances REST protein levels by modulating the activity of β-TRCP ubiquitin ligase. Importantly, Casein kinase 1 (CK1) is known to phosphorylate REST and enhance the β-TRCP activity leading to ubiquitin-mediated proteasomal degradation of REST. Therefore, treatment of SPINK1-positive 22RV1 cells with CK1 inhibitor resulted in restoration of REST protein levels, accompanied with reduced SPINK1 levels and its oncogenic properties (Tiwari et al., 2020), thus emphasizing the repressive role of REST protein in the regulation of SPINK1 and disease progression toward NE-like phenotype.

Evaluation of the transcription factors involved in lineage plasticity in prostate tumors showed SRY-box transcription factor 2 (SOX2) to be highly upregulated in tumors with altered TP53 and RB1 (TP53^Alt^, RB1^Alt^) compared to wildtype TP53 and RB1 (TP53^WT^, RB1^WT^) tumors. Furthermore, *SOX2* silencing in the LNCaP cells overexpressing AR with inactivated RB1 and TP53 reversed the increased expression of basal (CK5, CK14, and TP63) and neuroendocrine (SYP, CHGA, and NSE) lineage markers induced due to *TP53* and *RB1* loss (Mu et al., 2017). Furthermore, the role of SOX2 has been reported in repressing adenocarcinoma specific genes by enhancing the expression and activity of lysine-specific demethylase 1A (LSD1/KDM1A) (Li et al., 2019), highlighting the potential of SOX2 as a lineage reprogramming factor in neuroendocrine prostate tumors. Moreover, a neural specific transcript variant of LSD1 also known as LSD1+8a, has been shown to be exclusively expressed in NEPC tissue samples and patient-derived xenograft samples, and LSD1+8a/SRRM4 co-upregulated gene signature was found to be exclusively activated in aggressive NEPC patient tumors, that are different from those regulated by the canonical LSD1 (Coleman et al., 2020). A recent study reported Serine Peptidase Inhibitor, Kazal type 1 (SPINK1) to be transcriptionally repressed by AR and its corepressor REST, and androgen deprivation resulted in its upregulation. Furthermore, SOX2 was shown to modulate the expression of SPINK1 during the NE-transdifferentiation of LNCaP cells (Tiwari et al., 2020). This study also confirmed the role of SPINK1 in EMT, stemness and NE-transdifferentiation. Additionally, a subset of NEPC patients exhibit elevated levels of SPINK1, suggesting its role in the maintenance of the NE-like phenotype (Tiwari et al., 2020).

Metabolic reprogramming plays a crucial role in cancer progression and therapy-resistance (Hanahan and Weinberg, 2011). The ground-breaking discovery by Warburg suggested the preference of aerobic glycolysis over oxidative phosphorylation in cancer cells which primarily rely on the mitochondrial oxidative phosphorylation for adenosine 5'-triphosphate (ATP) generation. This resulted in the higher rate of glucose uptake and lactate production in presence of oxygen (Vander Heiden et al., 2009). Early clinical studies have shown that fluorodeoxyglucose (FDG)-PET imaging which is based on increased glucose uptake by cancer cells failed to detect naïve localized PCa (Effert et al., 1996), but can detect the advanced stage small cell prostate cancer (SCPC) (de Carvalho Flamini et al., 2010), highlighting the metabolic differences underlying the adenocarcinoma and SCPC. Moreover, the higher uptake of glucose has been associated with the elevated expression of Glucose Transporter 1 (GLUT1) in poorly differentiated hormone-independent PCa (Effert et al., 2004). It has also been observed that PCa switch to aerobic glycolysis only at the advanced stages of the disease progression and correlates with poor clinical outcomes (Pertega-Gomes et al., 2015). Of interest, the gene expression profile of NEPC patients showed glycolysis and lactic acid production as the most significantly upregulated pathways in these tumors (Choi et al., 2018). It has been shown that higher expression of the plasma membrane transporter monocarboxylate transporter 4 (MCT4) facilitated the enhanced secretion of lactic acid, while antisense oligonucleotides mediated silencing of *MCT4* led to reduced lactic acid secretion, glucose metabolism and NEPC cell proliferation (Choi et al., 2018). Recently, reduced PKCλ/ι has been reported in *de novo* and treatment-related NEPC differentiation, which resulted in upregulated mTORC1/ATF4/PHGDH and promoted serine biosynthesis, leading to increased S-adenosyl methionine (SAM). Moreover, higher mTORC1 activity, stronger nuclear ATF4 staining and increased expression of PHGDH was also detected in NEPC tumors compared to adenocarcinoma, suggesting the critical role of mTORC1/ATF4/PHGDH metabolic axis in increased cell proliferation and epigenetic reprogramming during NEPC development (Reina-Campos et al., 2019).

Numerous factors have been shown to be involved in maintaining the tumor cell plasticity (Table 1), however, more comprehensive in-depth studies are required to dissect the specific drivers which can be targeted for therapeutic implications.

**Table 1:** An overview of key molecular drivers involved in cell plasticity in the pathogenesis of prostate cancer.

**Molecular drivers**	**Regulatory mechanism**	**Phenotypic features**	**References**
**EPITHELIAL-MESENCHYMAL TRANSITION (EMT)**			
CDH1	Downregulated by Notch signaling	Silencing *CDH1* (E-Cad) promotes PCa cell migration, drug-resistance and metastasis	Wang et al., 2017; Loh et al., 2019
SOX9	Regulated by Wnt/β-catenin signaling	Enhances tumor cell proliferation and invasion	Wang et al., 2008
ZEB1	Shows bidirectional negative feedback loop with AR	Mediates androgen deprivation induced EMT	Sun et al., 2012
ZEB2	Differential regulation by AR	Potentiates cell invasion and migration	Jacob et al., 2014
SLUG	Androgen-responsive gene and AR coactivator	Facilitates PCa cell growth in androgen-deprived conditions	Wu et al., 2012
SNAIL	Transcriptionally repressed by AR	Plays a critical role in ADT induced epithelial-mesenchymal plasticity	Miao et al., 2017
CCL2	Silencing *AR* elevates CCL2 levels and STAT3 signaling	Promotes metastasis via macrophage recruitment	Izumi et al., 2013; Tsai et al., 2018
**STEMNESS**			
ABCG2	Membrane transporter found on prostate cancer stem cells	Maintain proliferative potential under hypoxic conditions, and efflux androgens	Huss et al., 2005
CD44	Cell-surface marker found on AR-independent basal prostate cells	CD44-positive PCa cells have high proliferative, clonogenic, tumorigenic, and metastatic potential	Liu et al., 1997; Patrawala et al., 2006
**THERAPY-INDUCED CELLULAR PLASTICITY**			
SNAIL	PEG10 regulates SNAIL expression via TGF-β signaling	Elevated levels found in tumor after castration in xenografts model and NEPC development	Akamatsu et al., 2015
ZEB1	Higher expression in castrated *PTEN* knockout mice and NEPC models	Induce stem cell-like properties and promotes androgen-independence in PCa	Li et al., 2014; Akamatsu et al., 2015
SOX11	Upregulated in *Pten* and *Trp53* inactivated mice model	Abiraterone treatment of *Pten*/*Trp53* inactivated mice lead to neuroendocrine differentiation	Zou et al., 2017
BRN2	AR repressed gene and regulates SOX2 expression	Key driver of aggressive tumor growth; higher levels found in NEPC compared to CRPC and adenocarcinomas	Bishop et al., 2017
**CELL-INTRINSIC FACTORS**			
AURKA	Amplified and overexpressed in NEPC	Functionally cooperate with N-MYC and drive neuroendocrine phenotype	Beltran et al., 2011; Lee et al., 2016
MYCN	Amplified and overexpressed in NEPC	Stabilizes AURKA, abrogates AR signaling, induces PRC2 silencing and serves as an oncogenic driver of NEPC	Beltran et al., 2011; Dardenne et al., 2016; Lee et al., 2016
EZH2	Highly expressed in advanced stage PCa and NEPC	Transforms the epigenetic landscape of PCa and NEPC	Varambally et al., 2008; Beltran et al., 2011; Clermont et al., 2015; Dardenne et al., 2016
REST	Downregulated in NEPC	Transcriptional corepressor of AR and implicated in NEPC development	Lapuk et al., 2012; Svensson et al., 2013; Tiwari et al., 2020
SOX2	Overexpressed in NEPC tumors consistent with RB1 and TP53 alterations	Required for lineage plasticity and antiandrogen resistance induced by inactivated RB1 and TP53	Bishop et al., 2017; Mu et al., 2017
SPINK1	Transcriptionally repressed by AR and REST and regulated by SOX2 in androgen deprived condition	Imparts cellular plasticity and maintenance of neuroendocrine phenotype	Tiwari et al., 2020
PKCλ/ι	Downregulated in NEPC	Its loss promotes serine biosynthesis, resulting in metabolic reprogramming to support cell proliferation and epigenetic changes	Reina-Campos et al., 2019
**CELL-EXTRINSIC FACTORS**			
TGF-β	Shows negative feedback loop with PMEPA1; cross talk with CXCR4; acts via both SMAD-dependent and independent pathways	Associated with PCa aggressiveness and bone metastasis	Derynck and Zhang, 2003; Bhowmick et al., 2004; Ao et al., 2007; Fournier et al., 2015
IL-6	Secreted by aggressive PCa cells	Elicits fibroblast activation and secrete MMPs	Giannoni et al., 2010
BMP6	Secreted by PCa cells and show feedback loop with IL-6	Upregulates IL-6 expression from macrophages, leading to neuroendocrine differentiation of PCa cells	Lee et al., 2011
IL-1 family genes	Secreted by prostate epithelial cells	Induce secretion of proinflammatory cytokines (CXCL-1,−2,−3 and IL-8) in stromal cells and facilitate cancer progression.	Kogan-Sakin et al., 2009
SPINK1	Regulated by NF-κB and C/EBP upon DNA damage in stromal cells	Serves as a senescence-associated secretory factor and a non-invasive biomarker of therapeutically damaged tumor microenvironment	Chen et al., 2018a

### Cell-Extrinsic Factors

The external cues along with the cell intrinsic factors, such as transcriptional and epigenetics regulation, are the key determinants for the tumor heterogeneity in PCa patients (Davies et al., 2018). The cell extrinsic factors constitute the tumor microenvironment which dictates the process of cellular plasticity in most of the malignancies including prostate (Yates, 2011). The concept of influence of microenvironment on tumor cells was initially proposed by an English surgeon, Stephen Paget, who laid the foundation that the conducive microenvironment is essential for the colonization of the disseminated tumor cells, also known as the seed and soil theory (Paget, 1889). The tumor microenvironment includes blood vessels, stromal cells namely, cancer-associated fibroblasts (CAFs), endothelial cells, neuroendocrine cells and infiltrating immune cells, growth factors and chemokines secreted by either tumor cells or stromal cells and many extracellular matrix proteins such as laminin, fibronectin, and collagen (Yates, 2011). Apart from the dynamic interaction between tumor and stromal cells, physical (elasticity and stiffness) and biochemical properties (protein composition) of the extracellular matrix (ECM), as well as access to nutrients and oxygen also governs the cellular plasticity of the tumor cells (Yates, 2011; Davies et al., 2018; Patel et al., 2019).

Among the different stromal cells, CAFs play a critical role in modulating the plasticity of the cancer cells. The CAFs are well-known to support tumor growth, resistance to therapy and metastasis by creating a tumor-promoting microenvironment for the cancer cells to proliferate, invade and evade the immune suppression (Cirri and Chiarugi, 2011). Moreover, CAFs mainly originate from the fibroblasts residing in tumor under the influence of the transforming growth factor (TGF-β) secreted by cancer cells (Massague, 2008; Bellomo et al., 2016). In addition, stromal cells such as pericytes or inflammatory cells may also transdifferentiate to CAFs via the process known as mesenchymal-to-mesenchymal transition (MMT) under the influence of TGF-β and other cytokines secreted in the tumor microenvironment (Bellomo et al., 2016). Similar to cancer cells, the CAFs also produce TGF-β which acts as an autocrine and paracrine factor and regulates the reorganization of the extracellular matrix and the interaction between tumor-stroma (Erdogan and Webb, 2017). Moreover, CAFs isolated from prostate carcinomas produce higher amounts of other cytokines namely, pro-inflammatory cytokines, interleukin-6 (IL6) and bone morphogenetic factor (BMP6), thereby promoting tumor progression (Doldi et al., 2015). There is a reciprocal interplay between CAFs and tumor cells, wherein tumor cells secrete IL6 and promotes CAFs to secrete matrix metalloproteases (MMPs) which in turn remodels the ECM, and further induces secretion of IL6 from tumor cells, thereby driving EMT. In addition, CAFs promote tumor forming ability and stemness when co-implanted with PCa cells in mice xenografts, and importantly, these tumor-repopulating cells were found to be CD44-positive and CD24-negative (Giannoni et al., 2010). The prostate stromal cells are also known to secrete proinflammatory and cancer-promoting chemokines such as CXCL-1, CXCL-2, CXCL-3, and interleukin (IL)-8, which are the key regulators of cellular plasticity, culminating in inflammation, and PCa progression (Kogan-Sakin et al., 2009). The prostatic CAFs also produce stromal glutamine as a result of epigenetic reprogramming and contribute to NE-transdifferentiation (Mishra et al., 2018). Interestingly, it has been known that genotoxic effect of chemo- and radiation therapies prompt stromal cells to produce SPINK1 as a secretory factor, which induces EGFR-mediated signaling and imparts chemoresistance in the adjacent prostate tumor cells (Chen et al., 2018a).

Tumor associated macrophages (TAMs) are also known to play important role in regulating cellular plasticity and NE-transdifferentiation. For instance, BPH-1 cells when co-cultured with THP-1 cells differentiated macrophages, led to increased expression of mesenchymal markers, such as N-Cad, Snail, and TGF-β2, and this phenotype was abrogated upon incubating with anti-TGF-β2 neutralizing antibody (Lu et al., 2012). Further, conditioned media collected from macrophages induce expression of NE-marker and parathyroid hormone-related peptide (PTHrP) in LNCaP and TRAMP-C2 cells. In this feedback loop, BMP6 secreted from the PCa cells induce production of IL6 from the macrophages, which in turn stimulates the NE-transdifferentiation of PCa cells (Lee et al., 2011).

**Figure 1:**
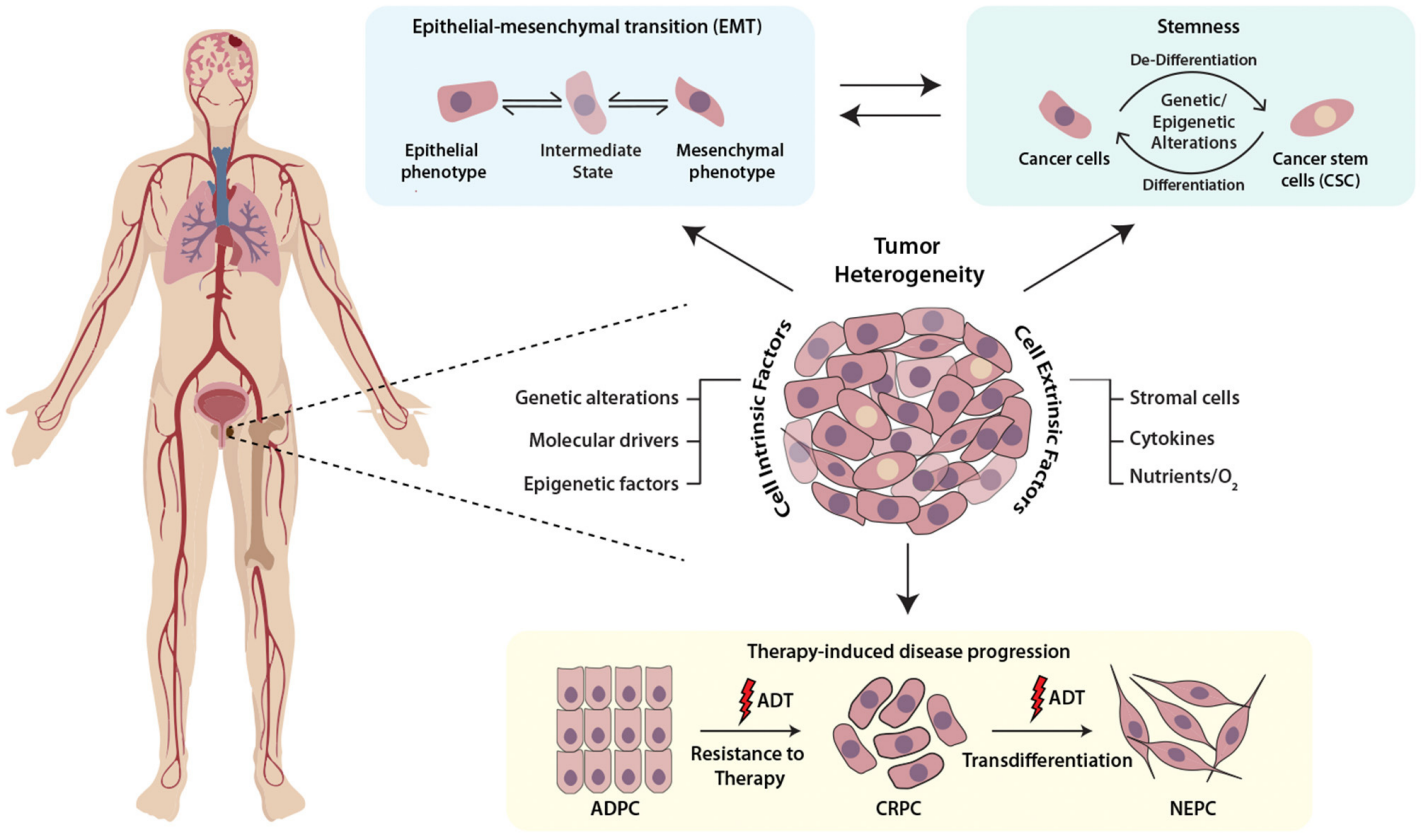
Schematic representing the multifaceted role of cellular plasticity in progression of prostate cancer. Prostate tumor comprises of heterogenous cell populations where both cell-intrinsic and -extrinsic factors confer cellular plasticity to enable transition between different cell fates by facilitating different mechanisms like epithelial-mesenchymal transition (EMT), stemness and drug resistance. Prostate tumor cell plasticity imparts resistance toward androgen deprivation therapy (ADT) during the progression of prostate adenocarcinoma (ADPC) to castrate-resistant prostate cancer (CRPC) stage, which may also transdifferentiate to neuroendocrine prostate cancer (NEPC).

Mounting evidence highlights the role of the physiochemical properties such as hypoxia or oxidative stress as key regulators of cellular plasticity in tumors. For instance, hypoxic stress leads to the upregulation of Hypoxia-inducible factor 1-α (HIF1α), which in cooperation with FOXA2, drives mesenchymal reprogramming and NE-transdifferentiation in PCa cells (Li et al., 2016). Another report indicates that hypoxia leads to reduced expression of transcriptional repressor REST, which in turn leads to hypoxia-induced neuroendocrine differentiation, followed by activation of associated AMPK pathway and autophagy (Lin et al., 2016). Multi-disciplinary approaches such as mathematical modeling and bioengineering tools, would allow fostering a hypoxic niche for exploring the events and mechanisms involved in adaptation of aggressive cancer behaviors, and would provide cues to disrupt the signaling pathways involved in crosstalk between cancer cells and tumor microenvironment (Figure 1).

## Targeting Cellular Plasticity and its Clinical Implications

Current studies are focused on targeting the markers and pathways involved in upholding the cellular plasticity in prostate cancer. Previous investigations have recommended the use of aurora kinase inhibitors in NMYC overexpressing prostate cancer, wherein it disrupts the N-Myc-AURKA complex and results in reduced tumor burden (Beltran et al., 2011; Dardenne et al., 2016; Lee et al., 2016). However, recent phase II clinical trials of AURKA inhibitor (NCT01799278), alisertib used for the treatment of metastatic NEPC patients showed efficacy in select cases (Beltran et al., 2019). Furthermore, N-Myc has been shown to cooperate with EZH2 and play critical role in changing the epigenetic landscape of AR and N-Myc target genes during NEPC transition. Elevated levels of N-Myc showed enhanced sensitivity to EZH2 catalytic SET domain inhibitor GSK503 in mice harboring N-Myc overexpressing 22RV1 xenografts (Dardenne et al., 2016). The EZH2 inhibitor (CPI-1205) combined with enzalutamide or abiraterone/prednisone are currently under phase Ib/II clinical trials (NCT03480646) for the treatment of metastatic CRPC cases. Recently, one of the homeobox transcription factors, ONECUT2 has been shown to synergize with hypoxia signaling in promoting NEPC transition. Importantly, hypoxia-activated pro-drug TH-302 showed remarkable reduction of the tumor growth in PDX models with higher levels of ONECUT2, suggesting it as a promising treatment strategy for NEPC (Guo et al., 2019). A recent study showed the therapeutic potential of rovalpituzumab tesirine (SC16LD6.5) in NEPC cases with higher expression of Delta-like ligand 3 (DLL3) (Puca et al., 2019). There is no direct effective therapy for targeting cellular plasticity, however, therapeutic modalities targeting the known molecular drivers of NEPC using small molecule inhibitors in combination with immune checkpoint inhibitors are under development (Table 2).

**Table 2:** Therapeutic interventions targeting key molecular drivers involved in the cellular plasticity of prostate cancer.

**Inhibitor**	**Target**	**Mechanism of action**	**Clinical trial and status**	**References**
Siltuximab (CNTO 328)	IL6	Chimeric monoclonal antibody which neutralizes IL6 and prevents STAT3 activation	NCT00401765; Completed	Hudes et al., 2013
Lycopene	IL6	Attenuates IL6 activity and abrogates STAT3 phosphorylation	NCT01949519; Completed	Tang et al., 2011
Apitolisib (GDC-0980)	PI3K and mTOR kinase	Inhibits PI3K-AKT-mTOR signaling axis	NCT00854152; Completed	Dolly et al., 2016
CRLX101	HIF1α	Nanoparticle drug-conjugate with camptothecin, inhibits HIF1α and DNA Topoisomerase I activity	NCT03531827; Active	Tian et al., 2017; Chen et al., 2018b
CPI-1205	EZH2	Cofactor-competitive inhibitor of wild type and mutant EZH2 catalytic activity	NCT03480646; Active	Taplin et al., 2019
GSK2816126	EZH2	Inhibits EZH2 activity and reduces global methylation of H3K27me3 marks	NCT02082977; Terminated	Yap et al., 2016
PF-06821497	EZH2	Selective inhibitor of EZH2 activity	NCT03460977; Active	Kung et al., 2018
ZEN003694	N-MYC	Inhibits BET proteins and dysregulates N-MYC-mediated transcriptional programming	NCT02705469; Completed	Schafer et al., 2020
Alisertib (MLN8237)	AURKA	Inhibits interaction between AURKA and N-MYC, thereby disrupts N-MYC mediated signaling	NCT01799278; Completed	Beltran et al., 2019
Rovalpituzumab Tesirine (SC16LD6.5)	DLL3	Antibody–drug conjugate targeting DLL3 (a Notch ligand)	NCT02674568; Completed	Puca et al., 2019

## Conclusion

**Figure 2:**
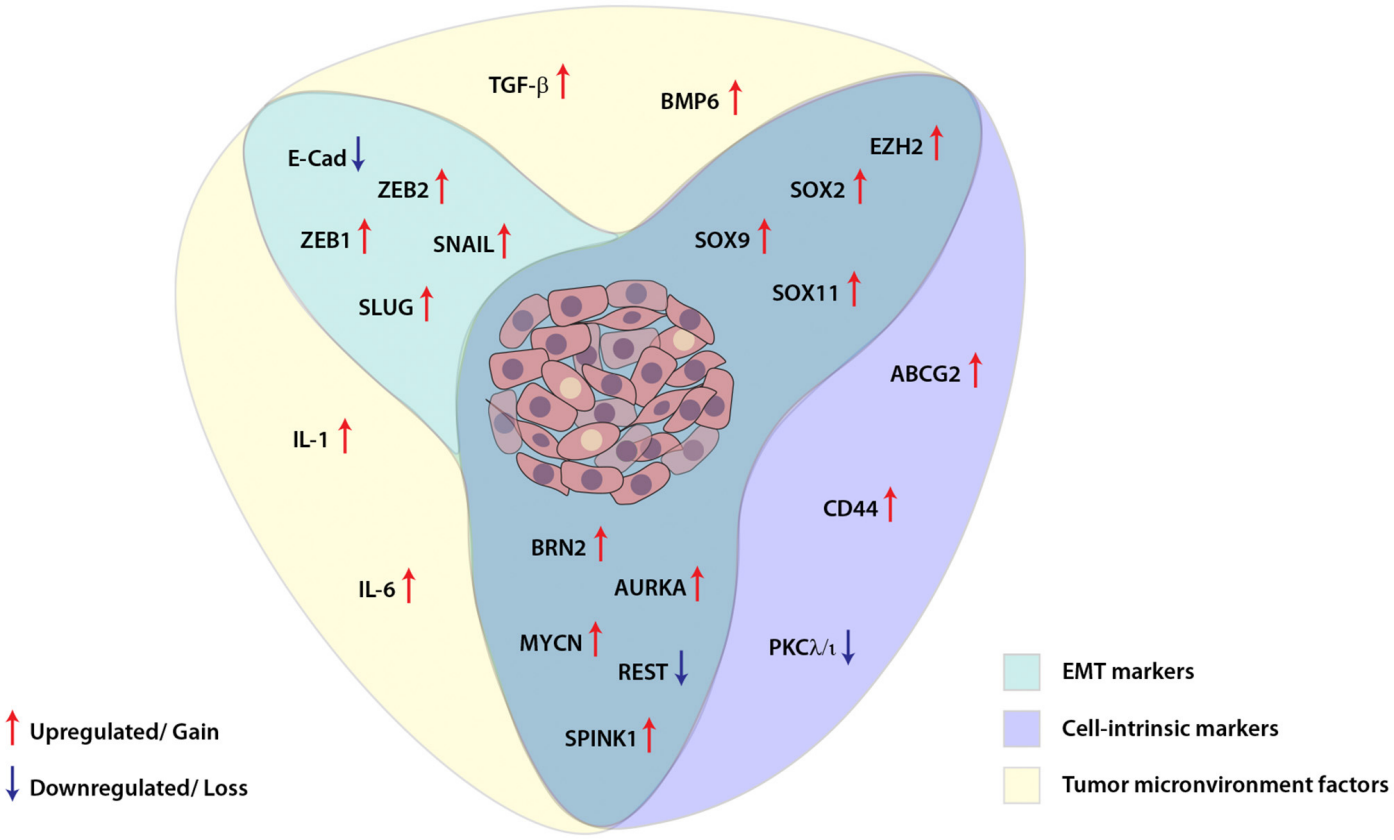
Schematic showing interplay between key molecular players involved in cellular plasticity in prostate cancer. Molecular markers associated with epithelial-mesenchymal transition (EMT), cell-intrinsic factors and tumor microenvironment are deployed for imparting plasticity in prostate cancer cells. These EMT and cell-intrinsic factors are regulated by cytokines and other growth factors released in the tumor microenvironment, which in turn are modulated by different transcription factors, transcriptional/post-transcriptional events and dysregulated signaling pathways in the cancer cells.

Similar to other malignancies, in prostate cancer as well, cellular plasticity is induced as a result of different contributing factors and governs a diverse set of characteristics which are involved in facilitating tumor dissemination, metastatic spread to distant sites and conferring resistance toward therapy (Figure 2). Despite the clinical benefits of ADT for the treatment of PCa, emerging evidence has suggested that ADT propels the cancer cells toward therapy-induced resistance and emergence of aggressive AR-independent variants of prostate cancer. Therefore, understanding the dynamics of tumor cell plasticity during transition from androgen responsive to androgen non-responsive state holds a prime importance in targeting the PCa progression. Also, in order to discover new therapeutic avenues enormous efforts are required to explore the underlying mechanisms involved in ADT mediated resistance or chemotherapeutic drug resistance of cancer cells in the clinical spectrum of prostate cancer stages. In conclusion, therapies against the cell plasticity, alone or in combination with AR-antagonists might prove effective for the clinical management of advanced stage CRPC or NEPC patients.

## Author Contributions

RT, NM, and BA reviewed the literature and contributed to manuscript writing. All authors contributed to the article and approved the submitted version.

## Conflict of Interest

The authors declare that the research was conducted in the absence of any commercial or financial relationships that could be construed as a potential conflict of interest.
